# Knowledge, Attitude, and Practices (KAP) on Antibiotic Use and Disposal Ways in Sidama Region, Ethiopia: A Community-Based Cross-Sectional Survey

**DOI:** 10.1155/2023/8774634

**Published:** 2023-07-26

**Authors:** Baye Sitotaw, Workneh Philipos

**Affiliations:** Bahir Dar University, Department of Biology, P.O. Box 79, Bahir Dar, Ethiopia

## Abstract

Antibiotic resistance has been among the top public health threats elsewhere. Scientific information on knowledge, attitudes, and practices (KAP) at the community level towards antibiotic use and disposal ways is a vital step for effective intervention. This study aimed at determining the levels of KAP and associated risk factors for antibiotics in and around Hawassa City, southern Ethiopia. A community-based cross-sectional study was conducted, and data were collected using a structured questionnaire. Descriptive statistics, chi-square test, and logistic regression were used to analyze and interpret the results. A total of 504 participants with a mean age of 35.32 ± 9.03 years were included in the study. Most of the participants were urban dwellers (59.5%); more than half (55.6%) of the participants were male; most of the participants (62.7%) were at least college graduates; about half were employed (52.4%); about 41.7% of the participants had a large family size (≥7) with a mean family size of 5.7 ± 2.7; the average family monthly income was ETB 7213.71 ± 3673, and over three-fourth (74.8%) of the study participants were married. In addition, about 83.13% of the study participants heard about antibiotics; almost all of them (99.8%) had ever used antibiotics at some point in their life (75% of which used antibiotics within 6 months), and all of them could name at least one common type of antibiotic. Moreover, most of the participants (86.5%) did not receive any training related to antibiotics, and 29.4% of them obtained antibiotics without a prescription. Most participants had poor knowledge (64%), negative attitudes (60.4%), and poor practices (55%) towards antibiotic use, resistance, and disposal methods. Significant and positive linear correlations between knowledge and attitude (*r* = 0.539, *P* ≤ 0.001), knowledge-practice (*r* = 0.532, *P* ≤ 0.001), and attitude-practice (*r* = 0.786, *P* < 0.001) were also observed. Most of the sociodemographic variables were significantly associated with the mean KAP scores of the study participants. Living in a rural area, having a large family size, and being female, married, illiterate, and farmer resulted in a very low level of knowledge. Similarly, living in a rural area, having a small family size, and being older and married resulted in a negative attitude. Furthermore, having a smaller family size, having a low family monthly income, and being married, illiterate, and self-employed resulted in poor practice. A very low level of KAP towards antibiotics among people living in and around Hawassa City calls for urgent and effective intervention strategies.

## 1. Introduction

Antibiotics are medicines that are used to treat several bacterial infections and help save lives when used appropriately [1]. They work by either stopping bacterial reproduction or directly destructing the cell components via certain specific mechanisms [2, 3]. Since the discovery of the first antibiotic, penicillin, several derivatives of penicillin and other varieties have been used to treat bacterial infections for centuries. Globally, antibiotic consumption rates have increased due to the concomitant increase in awareness of modern medications. It is estimated that antibiotic usage will double in 2030 [4]. Antibiotics should only be used on prescription by a physician following a proper diagnosis and should be disposed of following the standard protocols [5, 6]. In contrast, a considerable proportion of people distrust the proper use and disposal of antibiotics. Antibiotic misuse and improper disposal are widespread elsewhere, resulting in the emergence of antibiotic resistance pathogens at an alarming rate, posing a serious public health problem [7, 8]. It should be noted that the leftover or unused antibiotics must be returned to where they were purchased or obtained [9]. In case this is not possible, the unused antibiotics should be burned or boiled before dumping them. However, the majority of people in developing countries like Ethiopia have been practicing improper ways of antibiotic disposal [9].

Antibiotic resistance (ABR) occurs when bacteria develop the ability to defeat the drugs designed to kill them, which means that the bacteria continue to grow rather than being killed by the intended antibiotic. It should be noted that ABR develops naturally, but the big problem is that anthropogenic factors accelerate the process. When resistant pathogenic bacteria infect humans, it is very difficult or impossible to treat at all. Another serious issue is that resistant bacteria that are not pathogens can easily transfer this ability to other pathogenic bacterial strains. Antimicrobial resistance affects people at any stage of life and lifestyle, as well as the healthcare, veterinary, and agriculture industries. This makes it one of the world's most urgent public health problems [10, 11] and continues to be an urgent global public health threat, killing millions of people worldwide [12].

Antibiotic misuse (overuse, underuse, and/or unnecessary use) is the greatest risk factor for the emergence of ABR [7, 13, 14]. These and other causes of antimicrobial resistance are strongly linked to socioeconomic factors, and the risk factors in low-income countries are diverse and complex [15]. In low-income countries, there is a scarcity of data on the actual quantity of antibiotics being sold. The antibiotics are usually sold without any medical prescription or sold illegally. This favors the emergence of antibiotic-resistant bacteria [16, 17]. Several techniques are used to analyze the level of public understanding, perception, and practices regarding antibiotic use and disposal methods, either quantitatively or qualitatively. Knowledge, attitude, and practice (KAP) surveys are the most commonly used method for identifying misconceptions or misunderstandings that may be impediments to the activities we want to implement as well as potential barriers to behavior change.

Proper utilization of the prescribed antibiotics and disposal of the leftover ones are vital to controlling the emergence of ABR bacterial strains [7, 18]. This requires a community that has good knowledge, a positive attitude, and good practices (KAP) towards antibiotic use and disposal methods. Sadly, several studies have shown that a significant proportion of communities have low KAP in this regard [19–23]. The problem is especially acute in communities in developing countries, such as Ethiopia [14, 15, 19, 24]. There are numerous underlying causes for the low KAP level [25–28]. However, education level and age [18, 22, 29, 30], as well as other key socioeconomic characteristics such as income [26, 31, 32], are found to be the most important factors for the emergence of ABR. Most of the studies regarding KAP towards antibiotic use and related issues were institutional-based, whereas community-based assessment was scarce in Ethiopia.

The majority of previous studies on the knowledge, attitude, and practices (KAP) of antibiotic use and disposal methods were institution- or profession-based, i.e., KAP among animal farm owners [20, 30], hospitals and other health centers [33, 34], university students [33, 34], community pharmacies [35], and health professionals [36]. People in the twenty-first century are expected to demonstrate adequate knowledge of and practices regarding at least priority public health issues such as antibiotic resistance, regardless of profession, educational status, economic status, or geographical location. Relatively, only a few studies have attempted to address KAP among the general population (laypeople). In particular, community-based KAP surveys are limited in African countries, including Ethiopia [37].

Inappropriate antibiotic use is becoming highly prevalent in Ethiopia, and several studies have revealed the presence of many bacteria resistant to commonly used antibiotics [38–40]. Based on institutional studies, a high rate of ABR was also reported in Hawassa City [24, 41, 42]. There was no community-based KAP study on antibiotic use and related issues in and around Hawassa City. This study thus adds scientific information to the growing evidence that antibiotic misuse and related issues originate from Hawassa City. The purpose of this study was to assess the level of knowledge, attitudes, and practices regarding antibiotic use and disposal in Hawassa and its surrounding communities. This study provides valuable information to health professionals and other concerned bodies responsible for designing proper intervention strategies.

## 2. Materials and Methods

### 2.1. Description of the Study Area

This study was conducted in Hawassa City and its surroundings. Hawassa City is the capital city of the Sidama region and is situated on the eastern shore of Lake Hawassa inside the Great Rift Valley. It is 275 km south of Addis Ababa, the capital city of Ethiopia, and at an elevation of 1708 meters above sea level, at a latitude of 7.047845°N and a longitude of 38.479010°E. The area receives a mean annual rainfall of 1124 mm and has an average annual temperature of 20 to 25°C.

The city administration is divided into eight subcities and thirty-two kebeles (smallest administrative units). These eight subcities are Hayek Dare, Meneharia, Tabor, Misrak, Bahile Adarsh, Addis Ketema, Hawela-Tula, and Mehal Ketema. According to the Hawassa City Administration Finance and Economic Development Department, the city has 473,774 inhabitants, with 243,753 males and 230,021 females. There are one referral hospital, four government district hospitals, five private hospitals, seven health centers, 15 health posts, 51 private clinics, 46 drug stores, two diagnostic laboratories, and 55 pharmacies in the city administration [43].

### 2.2. Study Design, Sample Size Determination, and Sampling

A community-based cross-sectional study was conducted from February to August 2022 to assess the knowledge, attitude, and practices (KAP) towards antibiotic use, disposal, and other related issues using a structured and pretested questionnaire. The questionnaire was prepared first in English and then translated into two local languages (Amharic and Sidama languages). It was then back-translated to English to check the consistency. The questions were selected based on published literature studies and by assistance from health professionals.

Representative respondents were selected from different sites in Hawassa City and its surroundings using multistage sampling methods. First, the city was divided into urban and rural categories (strata). Then, three kebeles (the smallest administrative unit in Ethiopia) were randomly selected from each of the major categories. Finally, sampling points (households) were randomly selected within the three kebeles. The total sample size, indicated below, was distributed proportionally to each category.

The sample size was determined considering the 50% prevalence of low levels of KAP towards antibiotics use and disposal among communities in the study area, with a CI of 95% and a marginal error of 5%, giving a total sample size of 384. A single population proportion formula was used for sample size calculation as follows [44]:

(1) $$\begin{array}{c} n=\frac{z^{2}p\left(1-p\right)}{\alpha^{2}}=\frac{{\left(1.96\right)}^{2}*0.5\left(1-0.5\right)}{{\left(0.05\right)}^{2}}=384\text{,} \end{array}$$

where *n* is the sample size, *z* (critical value) is 1.96 at a confidence level of 95%, *α* is 0.05, and *P* (the sample proportion) is 50 (0.5).

The final sample size was 504 after adding a 5% nonrespondent rate and multiplying it by 1.25 for the design effect.

### 2.3. Source Population

The source population was people living in Hawassa City and its surrounding rural areas, Sidama Region, Ethiopia.

### 2.4. Study Population

The study population consisted of household members of the source population whose age was above 18 years.

### 2.5. Inclusion and Exclusion Criteria

Participants included in the study were residents of Hawassa City and its surrounding communities who had lived there for at least 6 months, were 18 years old or older, expressed a willingness to participate, and could speak, read, or write in English, Amharic, or Sidama. Participants who failed to fulfil all of the aforementioned inclusion criteria were excluded.

### 2.6. Study Variables

The study variables are sociodemographic variables as listed in Table 1, which are independent variables. The levels of knowledge, attitude, and practices (KAP) towards antibiotic use and disposal ways are the dependent variables.

### 2.7. Operational Definitions

Responses to the questions related to knowledge, attitude, and practices (KAP) were assessed using a scoring system. For dichotomous responses, the right response was given one score and the wrong response was given zero score (correct = 1 and wrong = 0). For questions having three options, a right response was given a score of 2; a neutral response, a score of one; and a wrong response, a score of zero (correct = 2, neutral = 1, and wrong = 0). Responses to the five-point Likert scale were given scores from 1 to 5 (strongly agree = 5, agree = 4, neutral = 3, disagree = 2, and strongly disagree = 1). All scores were summed to form a discrete variable and converted into a 100-point scale (%), taking the maximum possible score as 100%. Bloom's original cutoff points, with a slight modification, were used to judge knowledge as good (80%), moderate (60%–80%), or poor (>60%); attitude as positive (80%), moderate (60%–80%), or negative (>60%); and practices as good (80%), fair (60%–80%), or poor (>60%) [45]. The KAP levels were calculated first based on Bloom's cutoff points as good, moderate, and poor, and then to refine further, values below a mean score were given a level of very poor or very negative. For the sake of simplicity, the “neutral” and “I do not know” responses were considered as wrong responses for the information in Table 2, and multiple responses to each of the correct options were given one score, i.e., agree and strongly agree and disagree and strongly disagree were merged.

### 2.8. Data Collection and Quality Control

Data collectors (researchers and researcher assistants) were trained for two days on the data collection tools and techniques. Data from illiterate respondents =  were collected by interviewing them using the questions. The collected data were checked manually every day to check the completeness and consistency. Double data entry was performed by two separate individuals to crosscheck the data entry. A pretest of the questionnaire was conducted in the same place (in the study area) to ensure that the study participants understood what they were intended to know, and some modifications to the questions were made accordingly. Data collectors were supervised daily by principal investigators (research advisor) to ensure the collection of quality data. Moreover, a reliability test analysis was conducted to check the internal consistency of the instrument (questions).

Thirty-seven items (questions) were used to determine the level of KAP towards antibiotic role (5 items), antibiotic use (20 items), disposal ways (5 items), and resistance (8 items), and most of the questions were about the practices. The questions focus on the role of antibiotics or differentiating between viral and bacterial infections (6 items), the meaning and causes of antibiotic resistance (4 items), the correct use or misuse of antibiotics (22 items), and ways of antibiotic disposal (5 items). Fourteen items were used to determine the level of knowledge; seven items were used to determine the level of attitude, and 16 items were used to determine the level of practice. Cronbach's *α* was determined to check internal consistency, where values of 0.7 ≤ *α* < 0.9 are considered good and acceptable [46, 47].

### 2.9. Ethics Approval and Consent to Participants

Approval and ethical clearance were obtained from the Review Research Ethics Committee of the College of Science, Bahir Dar University, Ethiopia, with reference number PRCSVD/283/2014 E.C (2022 GC). The objectives and purpose of the study were clearly explained to the study subjects, and verbal informed consent was obtained before data collection. Participants were also informed that they had the right to not participate in the study. The information was kept confidential, and the data were collected anonymously throughout the study.

### 2.10. Data Analysis

The data were entered and processed in a Microsoft Excel spreadsheet for coding. The normality of the data and homogeneity (reliability) of variance for dependent variables within sampling sites were checked using the Kolmogorov–Smirnov test and Levene test, respectively. After that, the data were analyzed using the Statistical Package for Social Sciences (SPSS) 23.0. Descriptive statistics were used to determine frequency, percentage, mean, and standard deviation and are presented in tables. Then, the chi-square test was used to check whether there were significant associations between dependent and explanatory (independent) variables (risk factors). Statistical differences and associations were considered significant at *P* ≤ 0.05 and with a 95% confidence interval. Finally, logistic regression was performed to determine the degree of association between the dependent variable (level of KAP) and the independent variables. A univariate analysis (crude odds ratio, COR) was performed, and those independent variables giving *P* ≤ 0.25 were included in the equation to calculate the adjusted odds ratio (AOR) [48]. Because the level of knowledge of all participants was poor or very poor, the regression analysis was performed by comparing the poor level with a very poor level of knowledge.

## 3. Results

### 3.1. Sociodemographic Characteristics of the Participants

All selected participants (*n* = 504) returned the questionnaires (100% response rate). The sociodemographic characteristics of the participants are indicated in Table 1. Participants from urban sites (59.5%) outnumbered those from rural sites; the participants' ages ranged from 23 to 72 years, with a mean (±SD) of 37.27 (10.25); male participants slightly outnumbered females (55.6%); the majority of the participants (72.3%) worked in government institutions or nongovernmental organizations; more than half (52.6%) of the study participants were married; most (87%) were literate (at least completed primary school), and far more than half (64.7%) of the participants earned ETB 5000 per month.

In addition, about 83.13% of the study participants heard about antibiotics; almost all of them (99.8%) had ever used antibiotics at some point in their life (75% of which used antibiotics within 6 months), and all of them could name at least one common type of antibiotic. Moreover, most of the participants (86.5%) did not take any education related to antibiotic use and disposal methods, and about one-third (29.4%) of the participants obtained antibiotics without a prescription.

Seven questions (5 items from questions related to knowledge and 2 items related to attitudes) were removed from the analysis as these questions tend to decrease Cronbach's *α* value. After removing these items, Cronbach's *α* values were 0.800, 0.799, and 0.817 for knowledge, attitude, and practice items, respectively.

### 3.2. Level of Knowledge, Attitude, and Practices (KAP) towards Antibiotic Use, Disposal Ways, and Other Related Issues among the Participants

As indicated in Table 2, the overall percent score of KAP is 36 to 45, which is in a poor range (Tables 2 and 3). Participants demonstrated moderate knowledge only on two items (side effects of antibiotics (Q4) and antibiotics can kill bacteria (Q5)), while participants showed moderate practice on three items (Q8, Q10, and Q15), which are related to the use of the correct dose of antibiotics, consulting a doctor before taking antibiotics, and taking antibiotics on time according to the instructions.

Information in Table 3 was calculated based on the scoring method explained in the Data Analysis section. The KAP levels were calculated first based on Bloom's cutoff points as good, moderate, and poor, and then to refine further, values below a mean score were given a level of very poor or very negative. As shown in Table 3, all participants had poor or very poor knowledge of the role, use, or disposal methods of antibiotics; 78% had a negative or very negative attitude; and 63.3% had poor or very poor practices on antibiotic use and disposal methods. Correlations between knowledge, attitude, and practices were interpreted using the criteria suggested in [49] as 0–0.25 = weak correlation, 0.25–0.5 = fair correlation, 0.5–0.75 = good correlation, and greater than 0.75 = excellent correlation. Moreover, significant and positive linear correlations between knowledge-attitude (*r* = 0.539, *P*  <  0.001), knowledge-practice (*r* = 0.532, *P*  <  0.001), and attitude-practice (*r* = 0.786, *P*  <  0.001) were observed.

### 3.3. Association of the Level of KAP with Antibiotic Use and Disposal Ways among the Participants

As indicated in Table 4, except for family size, all sociodemographic variables were strongly associated with the level of knowledge. As stated earlier, all participants had poor or very poor levels of knowledge. When compared to other respective categories/levels, a high proportion of participants from rural areas (82.4%), older age (63%), females (65.6%), illiterate (96.9%), married (56.8%), farmers (97.1%), and low income (80%) had very poor levels of knowledge.

As indicated in Table 5, except for sex, all sociodemographic variables showed a strong association with the level of attitude, and most of the participants had negative attitudes. When compared to their counter categories, a high proportion of participants from rural areas (90.2%), older age (97.8%), illiterate (100%), married (92%), small family size (93.5%), farmers (100%), and low income (96–100%) had negative attitudes.

Sex and family size showed no association with the level of practices on antibiotic use and disposal ways. However, the other six sociodemographic variables showed a strong association with the level of practice, and most of the participants had poor practice (Table 6). In comparison with their counter categories, a high proportion of participants from rural areas (76.5%), older age (78.3%), illiterate (96.9%), married (74.8%), farmers (94.2%), and low income (93–96.2%) had poor practices.

### 3.4. Logistic Regression Analysis (LRA) of the Most Important Risk Factors for Low Level of KAP

The most important risk factors for the level of KAP among Hawassa City communities were identified using bivariate logistic regression analysis (BLRA) (Table 7). In the modeling process, a univariate analysis was first performed with a 0.25 level of significance to select the candidate variables for multivariate analysis [50]. Because the level of knowledge of all participants was poor or very poor, the regression analysis was performed by comparing the poor level with a very poor level of knowledge.

#### 3.4.1. Association of Antibiotic Use and Disposal with the Level of Knowledge

All of the eight variables were significant in the univariate analysis and included in the bivariate analysis. However, only residence, sex, family size, marital status, and occupation were found to be the most important predictors (*P* ≤ 0.05) of the level of knowledge among the study participants in Hawassa City and its surroundings (Table 7). Age, educational level, and family income also showed significant associations with the level of knowledge, but the effect was nonlinear.

As a result, very poor level of knowledge (VPLK) was increased by 5-fold (AOR = 5.45, CI = 2.78, 10.99; *P* ≤ 0.001) in participants who lived in rural areas compared to those in urban areas (Table 7), VPLK was 2 times (AOR = 2.12; CI = 1.08, 4.19; *P* ≤ 0.05) higher in female participants than in males; VPLK was increased by 4-fold (AOR = 3.74; CI = 1.55, 8.97; *P* ≤ 0.001) and 28-fold (AOR = 28.46; CI = 10.19, 79.49; *P* ≤ 0.001) in participants who had a family size of 4 to 6 and ≥ 7, respectively, than who had a small family size (1 to 3); VPLK was increased by almost 3-fold in married participants (AOR = 2.86; CI = 1.42, 5.79; *P* ≤ 0.01) than in single ones; VPLK was about 7 times (AOR = 6.99; CI = 2.94, 16.63; *P* ≤ 0.001) and 57 times (AOR = 57.26; CI = 2.78, 10.99; *P* ≤ 0.001), respectively, higher in self-employed and farmer participants than in those employed in government institution or NGO (Table 7).

#### 3.4.2. Association of Antibiotic Use and Disposal with the Level of Attitude

Only five variables, namely, residence, gender, age, family size, and marital status, were significantly associated (*p* 0.05) with the level of attitude (Table 8) and were found to be the most important predictors of the level of attitude among the study participants in Hawassa City and its surroundings.

Accordingly, negative attitude (NA) was increased by 3-fold (AOR = 2.97, CI = 1.46–6.05; *P* ≤ 0.05) in participants who lived in rural areas versus urban areas (Table 8); NA was increased by 8 times (AOR = 8.32; CI = 2.13, 32.40; *P* ≤ 0.01) in older participants (above 40 years old) versus young participants (18 to 29 years old); NA was increased by 30-fold (AOR = 33.86; CI = 11.09–85.87; *P* ≤ 0.001) and 5-fold (AOR = 5.23; CI = 2.46–11.11; *P* ≤ 0.001) in participants who had a family size of 1 to 3 and 4 to 6, respectively, than who had large family size (≥7); NA was increased by 23-fold in married participants (AOR = 23.26; CI = 10.44–51.85; *P* ≤ 0.01) compared to single ones (Table 8).

#### 3.4.3. Association of Antibiotic Use and Disposal with the Level of Practices

Seven variables were found to be significant in the univariate analysis and were included in the bivariate analysis. In the bivariate analysis, family size, level of education, marital status, and family income were significantly associated (*P* ≤ 0.05) with the level of practices (Table 9) and were found to be the most important predictors of the level of practices among the study participants in Hawassa City and its surroundings. Accordingly, poor practices (PPs) were increased by 34-fold (AOR = 34.09; CI = 11.02–105.43; *P* ≤ 0.001) and 4-fold (AOR = 28.46; CI = 10.19, 79.49; *P* ≤ 0.001) in participants who had a family size of 1 to 3 and 4 to 6, respectively, compared to those who had large family size (≥7); PPs was increased by 40 times (AOR = 40.35; CI = 14.64–111.18; *P* ≤ 0.001) in married participants than in single ones; PPs was increased by 8 times (AOR = 8.65; CI = 1.18–67.80; *P* ≤ 0.05) in illiterate participants compared to college or university graduates; PPs was 15 times (AOR = 15.43; CI = 4.30–49.14; *P* ≤ 0.001) higher in self-employed participants compared to those employed in government institutions or NGO; PPs was 6 times (AOR = 6.7; CI = 1.97–22.68; *P* ≤ 0.05), 58 times (AOD = 58.29; CI = 14.73–215.03; *P* ≤ 0.001), and 397 times (AOR = 397.79; CI = 56.22–2814.35; *P* ≤ 0.001) higher in participants who earned ETB 5000 to 7000, 3000 to 5000, and 1000 to 3000 compared to those who earned greater than ETB 10000 (Table 9).

## 4. Discussion

Undoubtedly, antibiotics have saved human lives for centuries. The problem is that the way antibiotics are used and disposed off. It is well understood that inappropriate use and wrong ways of disposal lead to complex public health problems, resulting in the emergence of ABR [10, 11]. A myriad of research has been conducted to gain insight into the major risk factors aggravating the emergence of ABR and found that low levels of public awareness, wrong perception, and other sociodemographic and economic factors are found to be the most important contributing factors [26, 27]. More information from different localities and settings is needed to extend our understanding of the risk factors for the low level of knowledge, attitude, and practices (KAP) towards antibiotic use and disposal methods. In addition, most studies were institution-based, while community-based surveys are also important to obtain the general public's level of KAP. With this in focus, this study was initiated to assess the level of KAP and some risk factors towards antibiotic use and disposal methods in Hawassa City (Southern Ethiopia) and its surrounding areas to get insight into the problems and recommend practical interventions to alleviate it.

### 4.1. Level of KAP among the Study Participants

Based on the results of this study, the participants had generally low levels of KAP (64% poor knowledge, 60.4% negative attitude, and 55% poor practices) towards antibiotic use, resistance, and disposal, implying that there is a high risk of the emergence of antibiotic resistance in the area (Table 2). A systematic review and meta-analysis on antibiotic use and resistance patterns in Ethiopia by Muhie [14] demonstrated a considerably high prevalence of inappropriate antibiotic use (49.2%) and self-antibiotic prescription (43.3%), which is nearly similar to our study results. The use of leftover antibiotics from a family member and immature discontinuation of antibiotics were also common problems in the current study area. Participants' scores (level of KAPs) on most of the individual items as well as the overall scores were very low, showing that participants did not even have an acceptable level of KAPs on most of the items (questions). The findings of this study were almost comparable with community-based studies in Nepal [32] and Tanzania [6]. On the contrary, the results of this study have shown a far lower level of KAP compared to a community-based assessment in eastern Ethiopia [19], Romania [50], Tanzania [24], northern Ethiopia [51], Indonesia [18], and northwest Ethiopia [21]. The differences could be attributed to the local variation or settings, and this can justify that studies from different localities are needed for a better understanding of the problem and to implement effective strategies on the basis of the local factors.

Regarding the individual items related to knowledge, more than 80% of the participants failed to correctly answer questions related to antibiotic (AB) disposal methods, as well as could not differentiate between viral and bacterial infections. This may result in the disposal of unused antibiotics into the environment and the misuse of antibiotics for viral infections, both of which are potential causes of ABR. It is obvious that antibiotics are prescribed for the treatment of bacterial infections and are not effective against viruses. Knowing whether an infection is bacterial or viral is thus vital. Unfortunately, a considerable number of people violate this fact. For instance, Nepal et al. [32] demonstrated a high degree (84.6%) of antibiotic misuse (i.e., for viral infection) in a community of Rupandehi District in Nepal; Karuniawati et al. [18] reported that 73.12% of respondents in their study in Indonesia stated that they believed that antibiotics could be used to treat viral infections, and a study by Jifar and Jifar [19] in eastern Ethiopia reported that a large number of respondents (83%) replied that antibiotics (ABs) speed up the recovery from most coughs and colds. Furthermore, 76% of participants in our study did not understand the importance of completing a full course of antibiotic treatment even after feeling better, and 73% were unaware of the dangers of overuse or overdose. It should be noted that antibiotic overuse or incorrect use is one of the factors that contribute to bacterial resistance development [52].

Similarly, more than 60% of the participants in our study had negative or wrong attitudes about correct disposal ways and the right prescription of antibiotics (ABs), which in turn could aggravate the aforementioned problem [35, 53]. Furthermore, more than 84% of participants in this study threw away antibiotics in the garbage bin, which is also an important risk factor for antibiotic resistance development [53]. Moreover, 50 to 65% of participants used leftover AB, shared AB with others, and did not pay attention to the instructions or labels; and about 76% of the participants did not complete the full course when taking AB. A similar study in Qatar among university students and their families demonstrated a higher rate of using AB without prescription (82%), compared to the figure in our study (29.4%) [54]. However, Aljayyousi et al. [54] reported a lower prevalence of sharing AB with family members or friends (37%), failing to complete a full course of AB treatment (45%), and consuming leftover AB (27%) compared to the results of this study (Table 2). Similarly, a study in Romania by Pogurschi et al. [55] reported that only 29% of the study participants failed to complete the full course of AB treatment, which is still better than our study results. The variations between our study results and other studies could be attributed to the social settings including socioeconomic, cultural, and demographic variables as demonstrated by extensive studies by Hassen et al. [56]. All of these attributes are potential factors for the emergence of ABR. Stopping the medication before the full course was found to have a high prevalence (76%), which is one of the major factors contributing to the bacteria becoming resistant to future treatments. The ones that survive will have had some exposure to the antibiotic and may consequently develop resistance to it. It is strongly recommended that an individual complete the course of antibiotic treatment even if symptoms have improved.

### 4.2. Associated Risk Factors for the Low Level of KAP among the Study Participants

In this study, eight sociodemographic characteristics of the participants, namely, residence type, gender, age, marital status, family size, educational status, occupation, and monthly income, were considered as independent variables that may affect the level of KAP of the participants. Even though the patterns could vary from place to place and between different community settings, sociodemographic variables are generally known to affect the level of KAP towards antibiotic use, disposal methods, and related issues [14–23, 27–34, 36, 37, 51, 55, 57].

Based on the chi-square test, all sociodemographic characteristics, except that of family size, showed a significant association with the level of knowledge. It is well documented that sociodemographic factors highly influence the level of knowledge regarding antibiotic use and related issues [18, 30, 56, 58], and the result of this study is thus in line with previous studies. Regarding attitude, all sociodemographic factors, except for the sex of the respondents, were significantly associated with the level of attitude. A study by Alfirani et al. [59] also reported strong associations between attitudes and sociodemographic factors, such as age and educational level, towards antibiotic use and disposal methods. Furthermore, the level of practice was significantly associated with six of the eight sociodemographic characteristics, whereas sex and family size were not significantly associated with the level of practice. The most important component of KAP is practice, as the ultimate goal is to have a community that practices appropriate actions (acts on) in the daily activities. Practice is usually positively influenced by the knowledge and attitude of the individual [21, 30, 58].

### 4.3. Determining the Most Important Factors for KAP Using Logistic Regression

Antibiotic misuse and incorrect disposal practices are widespread problems [7, 60] and pose one of the most serious public health problems, i.e., antibiotic resistance [11]. The most important factors for the aforementioned problems have been identified as a community's sociodemographic characteristics [26, 56, 58]. In this study, the degree of association of the level of KAP with antibiotic use, resistance, and disposal methods varied among the predictor variables. Accordingly, the most important risk factors for the very low level of knowledge observed in this study were found to be residence, gender, family size, marital status, level of education, and occupation of the participants. Residents of rural areas, females, participants with large family sizes, married, illiterate, and farmer participants had a very low level of knowledge compared to the other respective categories. Educational level, which is a very important sociodemographic factor, has been reported as an important predicting factor for the level of knowledge on antibiotic use and resistance in community-based studies by Karuniawati et al. [18], Gebeyehu et al. [20], Sindato et al. [30], Hassan et al. [56], and Di et al. [58], which are in line with our results. Regarding the gender, Simegn et al. [36] also reported that females had poor knowledge than males, which is in line with our result. It may not be a surprise that people living in rural areas, females, and farmers had insufficient knowledge as they are often marginalized in developing countries. In this study, unmarried (singles) and participants with small family sizes had better knowledge, which is likely linked to the low social burden on them.

Similarly, the most important risk factors for the negative attitude were found to be residence, age, family size, and marital status of the participants. Participants living in rural areas, older age groups, small family sizes, and married had relatively poor or negative attitudes compared to their counter categories. Furthermore, the most important predictors of the low level of practice were found to be family size, marital status, educational level, and occupation of the participants. Participants with smaller family sizes, married, illiterate, low family monthly income, and self-employed had poor practices compared to their counter categories. In agreement with our results, the educational level and occupation of the participants have been found to be important predictors of the level of practices on antibiotic use and resistance [28, 58].

## 5. Conclusions and Recommendations

This study demonstrated a very low level of knowledge, attitude, and practices (64% poor knowledge, 60.4% negative attitude, and 55% poor practices) on antibiotic use and disposal ways among people living in and around Hawassa City, southern Ethiopia. Among the sociodemographic variables considered in this study, residence, gender, family size, marital status, level of education, and occupation of the participants were found to be the most important factors contributing to the low level of KAP in the study population. It is strongly advised to raise public awareness and practices regarding antibiotic use and disposal methods in order to control the development of antibiotic resistance and subsequent public health burdens.

## 6. Limitations of the Study

We are aware that the study is limited as it did not include other data collection tools such as focus group discussions and conduct in-depth interviews with key informants in the community, as well as did not consider other antimicrobials (antifungal, helminth, and malaria drugs) for a comprehensive understanding of how medicines are used and disposed of in the community. The rural community could have also been sampled farther from Hawassa City to see a clear discrepancy in the level of KAP between rural and urban communities in the area. Another important issue is that, in such studies, self-reported bias (self-administered questionnaire) may affect the quality of the information. We are also aware that there might be a gap between what is said and what is done in the KAP survey, and such a survey reveals what is said but cannot be sure about what was done.

## Figures and Tables

**Table 1:** Sociodemographic characteristics and some other information about study participants in and around Hawassa City (*n* = 504), 2022.

Variables	Categories	Frequency *N* (%)
Place of residence	Urban	300 (59.5)
	Rural	204 (40.5)

Age of the respondent (years)	18–29	141 (28)
	30–39	225 (44.6)
	≥40	138 (27.3)

Sex of the respondent	Male	280 (55.6)
	Female	224 (44.4)

Educational status of the respondent	No education	65 (12.9)
	Primary or secondary school completed	122 (24.2)
	College or university graduate	317 (62.9)

Marital status of the family head	Married	377 (74.8)
	Single	127 (25.2)

Family size	1–3	139 (27.6)
	4–6	155 (30.8)
	≥7	210 (41.7)

Occupation of the family head	Employed	265 (52.6)
	Self-employed	135 (26.8)
	Farmer	104 (20.6)

Family monthly income in ETB^*∗*^	≥10000	90 (17.9)
	7000–10000	171 (33.9)
	5000–7000	65 (12.9)
	3000–5000	98 (19.4)
	1000–3000	80 (15.9)

Have you ever heard about antibiotics	Yes	419 (83.13)
	No	85 (16.9)

Have you ever used antibiotics?	Yes	503 (99.8)
	No	1

Type of antibiotics commonly taken and listed by the participants	Amoxicillin	193 (38.3)
	Ampicillin	32 (6.3)
	Azithromycin	53 (10.5)
	Cephalexin	17 (3.4)
	Ciprofloxacin	120 (23.8)
	Dicloxacillin	34 (6.7)
	Doxycycline	20 (4.0)
	Gentamicin	7 (1.4)
	Metronidazole	26 (5.2)
	Vancomycin	2 (0.4)

When did you last use antibiotics?	≤6 months	378 (75)
	6 to 12 months	63 (12.5)
	1 to 2 years	26 (5.2)
	≥2 years	37 (7.3)

Are you currently taking antibiotics?	Yes	185 (36.7)
	No	319 (63.3)

Where did you often get the antibiotics?	Clinic	177 (35.1)
	Health center	72 (14.3)
	Hospital	107 (21.2)
	Pharmacy without prescription	148 (29.4)

Have you ever been in any educational campaign regarding antibiotic use and disposal?	Yes	68 (13.5)
	No	436 (86.5)

^*∗*^1 USD = ETB 55.

**Table 2:** Knowledge, attitude, and practices (KAP) of participants (*n* = 504) at Hawassa City and the surrounding area, 2022.

Item	Description	Percentages of correct answer
*Items related to knowledge; Cronbach's α value* *=* *0.80 after some items removed*		
Q1	Can you differentiate between bacterial and viral infection?	Yes (44)
Q2	Antibiotic resistance means that bacteria will not be killed by antibiotics	Yes (47.6)
Q3	Infections caused by antibiotic-resistant bacteria cannot be easily cured or cannot be cured	Yes (42.9)
Q4	Antibiotics have no side effect	No (61.7)
Q5	Antibiotics can kill bacteria	Yes (76)
Q6	Antibiotics can kill viruses^*∗*^	No (10.1)
Q7	Antibiotics are prescribed for most cough and cold^*∗*^	No (17.1)
Q8	Antibiotics are effective for most sore throat^*∗*^	No (9.7)
Q9	Burning unused or leftover antibiotics is necessary	Yes (21)
Q10	Throwing expired antibiotics into the garbage does not have negative effects^*∗*^	No (8.1)
Q11	If antibiotics are taken for a long period of time, bacteria may become resistant to antibiotics	Yes (58.5)
Q12	If antibiotics are taken less than the prescribed dose, bacteria may become less resistant to antibiotics^*∗*^	No (10.1)
Q13	If twice the prescribed dose of antibiotics is taken, the effects of antibiotics will be more rapid	No (26.8)
Q14	The prescribed dose and duration of antibiotics can be terminated if the symptoms improve	No (24)
	Overall	36.05

*Items related to attitudes; Cronbach's α value* *=* *0.799*		
Q1	I expect medicine to be prescribed by my doctor if I suffer from diseases symptoms	Yes (55.3)
Q2	I believe that antibiotics cure my cold faster^*∗*^	No (16.3)
Q3	I prefer to burn unused or expired antibiotics	41.5
Q4	I prefer to keep the expired antibiotics deep into the soil	41.3
Q5	We can buy antibiotic from medicine shops/pharmacies directly	40.7
Q6	We can use antibiotics after the suggestions from friends/neighbors	40.9
Q7	Even if I have knowledge on the appropriate use of antibiotics, I often ignore the knowledge and follow the practice I used to do^*∗*^	41.3
	Overall	39.61

*Items related to practices; Cronbach's α value* *=* *0.817*		
Q1	I take antibiotics according to the instructions on the leaflet or labeled	Yes/agree (51.8)
Q2	I check if the right antibiotics are included in the prescribed medicine	Yes/agree (44)
Q3	I stop taking the prescribed antibiotics once I get better	No/disagree (45.5)
Q4	If my family member is sick, I usually give my prescribed antibiotic to them	No/disagree (47.4)
Q5	If I catch a cold, I ask for an antibiotic prescription to prevent my symptoms from getting worse	No/disagree (34)
Q6	I take leftover antibiotics when I have flu or other infections/omitted in raw data papers/questionnaires	No/disagree (45.4)
Q7	I normally keep antibiotic stock at home in case of emergency	No/disagree (41.7)
Q8	Do you consult a doctor before starting an antibiotic?	Yes (60)
Q9	Do you follow the advertisement (leaflets/Internet, etc.) while purchasing antibiotics?	Yes (42.9)
Q10	Do you take antibiotics on time according to the instructions	Yes (67.3)
Q11	Do you stop taking antibiotics without completing the full course?	No (37.7)
Q12	Have you ever used leftover antibiotics?	No (39.1)
Q13	I sometimes share my antibiotics with my family members when we have similar symptoms/illnesses	No (41.1)
Q14	I will stop taking my antibiotics once the illness/symptom improves	No (32.9)
Q15	I sometimes take antibiotics with lower dosage/frequency than the recommended dose	No (76.6)
Q16	I throw the leftover or unused antibiotics into garbage	No (15.7)
	Overall average	45.19

^*∗*^These items are removed from regression analysis.

**Table 3:** Level of knowledge, attitude, and practices among the participants (*n* = 504), 2022.

Factors	Good	Moderate/fair	Poor/negative	Very poor/very negative
Knowledge	—	—	46.7	52.2
Attitude	—	22	25.2	52.8
Practices	—	40.7	13.5	45.8

Values are in percentage (100-point scale).

**Table 4:** Association of the level of knowledge towards antibiotic use, disposal ways, and other related issues among the participants (*n* = 504), 2022.

Variables	Categories	Frequency *N* (%)	Level of knowledge		*χ* ^2^ (*P* value)
			Poor	Very poor	
Place of residence	Urban	300 (59.5)	205 (68.3)	95 (31.7)	125.02 (<0.001)
	Rural	204 (40.5)	36 (17.6)	168 (82.4)	

Age of the respondent (years)	18–29	141 (28)	98 (69.5)	43 (30.5)	37.42 (<0.001)
	30–39	225 (44.6)	92 (40.9)	133 (59.1)	
	≥40	138 (8.5)	51 (37)	87 (63)	

Sex of the respondent	Male	280 (55.6)	164 (58.6)	116 (41.4)	29.20 (<0.001)
	Female	224 (44.4)	77 (34.4)	147 (65.6)	

Educational status of the respondent	No education	65 (12.9)	2 (3.1)	63 (96.9)	194.58 (<0.001)
	Primary or secondary school completed	122 (24.2)	12 (9.8)	110 (60.2)	
	College or university graduate	317 (62.8)	227 (71.6)	90 (28.4)	

Marital status of the family head	Married	377 (74.8)	163 (43.2)	214 (56.8)	12.58 (<0.001)
	Single	127 (25.2)	78 (61.4)	49 (38.6)	

Family size	1–3	139 (27.6)	74 (53.2)	65 (46.8)	3.34 (>0.050)
	4–6	155 (30.8)	66 (42.6)	189 (57.4)	
	≥7	210 (41.7)	101 (48.1)	109 (51.9)	

Occupation of the family head	Employed	295 (52.6)	194 (73.2)	71 (26.8)	165.15 (<0.001)
	Self-employed	135 (26.8)	44 (32.6)	91 (67.4)	
	Farmer	104 (20.6)	3 (2.9)	101 (97.1)	

Family monthly income in ETB	≥10000	90 (17.9)	55 (61.1)	35 (38.9)	70.66 (<0.001)
	7000–10000	171 (33.9)	114 (66.7)	57 (33.3)	
	5000–7000	65 (12.9)	28 (43.1)	37 (56.9)	
	3000–5000	98 (19.4)	28 (28.6)	70 (71.4)	
	1000–3000	80 (15.9)	16 (20)	64 (80)	

**Table 5:** Association of the level of attitude towards antibiotic use, disposal methods, and other related issues among the participants (*n* = 504), 2022.

Variables	Categories	Frequency *N* (%)	Level of attitude		*χ* ^2^ (*P* value)
			Fair	Negative	
Place of residence	Urban	300 (59.5)	91 (30.3)	209 (69.7)	29.80 (<0.001)
	Rural	204 (40.5)	20 (9.8)	184 (90.2)	

Age of the respondent (years)	18–29	141 (28)	63 (44.7)	78 (55.3)	74.34 (<0.001)
	30–39	225 (44.6)	45 (20)	180 (80)	
	≥40	138 (8.5)	3 (2.2)	135 (97.8)	

Sex of the respondent	Male	280 (55.6)	55 (19.6)	225 (80.4)	2.08 (>0.05)
	Female	224 (44.4)	56 (25)	168 (75)	

Educational status of the respondent	No education	65 (12.9)	0	65 (100)	68.72 (<0.001)
	Primary or secondary school completed	122 (24.2)	4 (3.3)	118 (96.7)	
	College or university graduate	317 (62.8)	107 (33.8)	210 (66.2)	

Marital status of the family head	Married	377 (74.8)	29 (7.7)	348 (92.3)	178.93 (<0.001)
	Single	127 (25.2)	82 (64.6)	145 (35.4)	

Family size	1–3	139 (27.6)	9 (6.5)	130 (93.5)	36.92 (<0.001)
	4–6	155 (30.8)	31 (20)	124 (80)	
	≥7	210 (41.7)	71 (33.8)	139 (66.2)	

Occupation of the family head	Employed	265 (52.6)	76 (28.7)	189 (71.3)	138.38 (<0.001)
	Self-employed	135 (26.8)	35 (25.9)	100 (74.1)	
	Farmer	104 (20.6)	0	104 (100)	

Family monthly income in ETB	≥10000	90 (17.9)	57 (63.3)	33 (36.7)	142.80 (<0.001)
	7000–10000	171 (33.9)	48 (26.9)	125 (73.1)	
	5000–7000	65 (12.9)	5 (7.7)	60 (92.3)	
	3000–5000	98 (19.4)	0	98 (100)	
	1000–3000	80 (15.9)	3 (3.8)	77 (96.2)	

**Table 6:** Association of the level practices towards antibiotic use, disposal ways, and other related issues among the participants (*n* = 504), 2022.

Variables	Categories	Frequency *N* (%)	Level of practices		*χ* ^2^ (*P* value)
			Moderate	Poor	
Place of residence	Urban	300 (59.5)	157 (52.3)	143 (47.7)	41.75 (<0.001)
	Rural	204 (40.5)	48 (23.5)	156 (76.5)	

Age of the respondent (years)	18–29	141 (28)	86 (61)	55 (39)	44.74 (<0.001)
	30–39	225 (44.6)	89 (39.6)	136 (60.4)	
	≥40	138 (8.5)	30 (21.7)	108 (78.3)	

Sex of the respondent	Male	280 (55.6)	118 (42.1)	182 (57.9)	0.58 (>0.05)
	Female	224 (44.4)	87 (38.8)	137 (61.2)	

Educational status of the respondent	No education	65 (12.9)	2 (3.1)	63 (96.9)	124.40 (<0.001)
	Primary or secondary school completed	122 (24.2)	15 (12.3)	107 (87.7)	
	College or university graduate	317 (62.8)	188 (59.3)	129 (40.7)	

Marital status of the family head	Married	377 (74.8)	95 (25.2)	282 (74.8)	148.49 (<0.001)
	Single	127 (25.2)	110 (86.6)	17 (13.4)	

Family size	1–3	139 (27.6)	50 (36)	89 (64)	4.60 (>0.05)
	4–6	155 (30.8)	58 (37.4)	97 (62.6)	
	≥7	210 (41.7)	97 (46.2)	113 (53.8)	

Occupation of the family head	Employed	265 (52.6)	155 (58.5)	110 (41.5)	91.02 (<0.001)
	Self-employed	135 (26.8)	44 (32.6)	91 (67.4)	
	Farmer	104 (20.6)	6 (5.8)	98 (94.2)	

Family monthly income in ETB	≥10000	90 (17.9)	78 (86.7)	12 (13.3)	202.22 (<0.001)
	7000–10000	171 (33.9)	101 (59.1)	70 (40.9)	
	5000–7000	65 (12.9)	17 (26.2)	48 (73.8)	
	3000–5000	98 (19.4)	6 (6.1)	92 (93.9)	
	1000–3000	80 (15.9)	3 (3.8)	77 (96.2)	

**Table 7:** Logistic regression analysis of the explanatory variables against knowledge of the participants (*n* = 504), 2022.

Risk factors	Level of knowledge		Total (%)	COR (95% CI)	*P* value	AOR (95% CI)	*P* value
	Poor (%)	Very poor (%)					
*Residence*							
Urban	205 (68.3)	95 (31.7)	300 (59.52)	1			
Rural	36 (17.6)	168 (82.4)	204 (40.47)	10 (6.52–15.55)	<0.001	5.53 (2.78–10.99)	<0.001

*Gender*							
Male	164 (58.6)	116 (41.4)	280 (55.55)	1		1	
Female	77 (34.4)	147 (65.6)	224 (44.44)	2.67 (1.87–3.88)	<0.001	2.12 (1.08–4.19)	<0.05

*Age*							
18 to 29	98 (69.5)	43 (30.5)	141 (28)	1	<0.001	1	
30 to 39	92 (40.9)	133 (59.1)	225 (44.6)	3.295 (2.11–5.15)		3.67 (1.85–7.31)	<0.001
40 and above	51 (37)	87 (63)	138 (27.4)	3.88 (2.36–6.39)		1.10 (0.41–2.94)	>0.05

*Family size*							
1 to 3	74 (53.2)	65 (46.8)	139 (27.57)	1		1	
4 to 6	66 (42.6)	89 (57.4)	155 (30.75)	1.53 (0.96–2.43)	>0.05	28.46 (10.19–79.49)	<0.001
7 and above	101 (48.1)	109 (51.9)	210 (41.7)	1.23 (0.80–1.88)	>0.05	3.74 (1.55–8.97)	<0.01

*Marital status*							
Married	163 (43.2)	214 (56.8)	377 (74.80)	1	<0.001	2.86 (1.42–5.79)	<0.01
Single	78 (61.4)	49 (38.6)	127 (27.20)	0.48 (0.32–0.72)		1	

*Educational level*							
No education	2 (3.1)	63 (96.9)	65 (9.92)	79.45 (19.4–331)	<0.001	45.38 (7.28–282.9)	<0.001
High or primary school	12 (9.8)	110 (90.2)	122 (24.21)	23.12 (12.41–44.02)	<0.001	2.71 (0.91–8.06)	>0.05
Graduate	227 (71.6)	90 (28.4)	317 (62.89)	1		1	

*Occupation*							
Govt. or NGO employed	194 (73.2)	71 (26.8)	265 (52.6)	1		1	
Self-employed	44 (32.6)	91 (67.4)	135 (26.8)	5.65 (3.60–8.72)	<0.001	6.99 (2.94–16.63)	<0.001
Farmer	3 (2.9)	101 (97.1)	104 (20.6)	95.20 (28.26–288.37)	<0.001	57.26 (2.78–10.99)	<0.001

*FMI*							
10000 and above	55 (61.1)	36 (38.9)	90 (17.9)	1		1	
7000 to 10000	114 (66.7)	57 (33.3)	171 (33.9)	0.78 (0.46–1.33)	>0.05	1.97 (0.37–10.47)	>0.05
5000 to 70000	28 (43.1)	37 (56.9)	65 (12.9)	2.07 (1.08–3.09)	<0.05	0.45 (0.17–1.22)	>0.05
3000 to 5000	28 (28.6)	70 (71.4)	98 (19.4)	3.92 (2.13–722)	<0.001	0.5 (0.19–1.33)	>0.05
1000 to 3000	16 (20)	64 (80)	80 (15.9)	6.28 (3.14–12.56)	<0.001	0.33 (0.14–0.76)	<0.05

**Table 8:** Logistic regression analysis of explanatory variables on the attitude of the participants (*n* = 504), 2022.

Risk factors	Level of attitude		Total (%)	COR (95% CI)	*P* value	AOR (95% CI)	*P* value
	Moderate (%)	Poor (%)					
*Residence*							
Urban	91 (30.3)	209 (69.7)	300 (59.5)	1		1	
Rural	20 (9.8)	184 (90.2)	204 (40.5)	4.00 (2.37–6.75)	<0.001	2.97 (1.46–6.05)	<0.05

*Gender*							
Male	55 (19.6)	225 (80.4)	280 (55.6)	1		1	
Female	56 (25)	168 (75)	224 (44.4)	0.73 (0.48–1.12)	0.15	0.58 (0.29–1.17)	0.131

*Age*							
40 and above	3 (2.2)	135 (97.8)	138 (27.4)	36.34 (11.04–119.62)	<0.001	8.32 (2.13–32.40)	<0.01
30 to 39	45 (20)	180 (80)	225 (44.6)	3.23 (2.03–5.14)	<0.001	0.86 (0.42–1.77)	0.68
18 to 29	63 (44.7)	78 (55.3)	141 (28)	1		1	

*Family size*							
1 to 3	9 (6.5)	130 (93.5)	139 (27.6)	7.37 (3.54–15.36)	<0.001	30.86 (11.09–85.87)	<0.001
4 to 6	31 (20)	124 (80)	155 (30.8)	2.04 (1.25–3.32)	<0.05	5.23 (2.46–11.11)	<0.001
7 and above	71 (33.8)	139 (66.2)	210 (41.7)	1		1	

*Marital status*							
Married	29 (7.7)	348 (92.3)	377 (74.8)	21.86 (12.93–36.96)	<0.001	23.26 (10.44–51.85)	<0.001
Single	82 (64.6)	45 (35.4)	127 (25.2)	1		1	

**Table 9:** Logistic regression analysis of the explanatory variables on practices of the participants (*N* = 504), 2022.

Risk factors	Level of practices		Total *N*(%)	COR (95% CI)	*P* value	AOR (95% CI)	*P* value
	Moderate *N*(%)	Poor *N*(%)					
*Residence*							
Urban	157 (52.3)	143 (47.7)	300 (59.5)	1		1	
Rural	48 (23.5)	156 (76.5)	204 (40.5)	3.56 (2.40–5.29)	<0.05	0.45 (0.2–1.01)	0.054

*Age*							
40 and above	30 (31.7)	108 (78.3)	138 (27.4)	5.63 (3.32–9.53)	<0.001	1.41 (0.48–4.10)	0.52
30 to 39	89 (39.6)	136 (60.4)	225 (44.6)	2.39 (1.55–3.67)	<0.001	1.25 (0.59–2.67)	0.09
18 to 29	86 (61)	55 (39)	141 (28)	1		1	

*Family size*							
1 to 3	50 (36)	89 (64)	139 (27.6)	1.52 (0.98–2.37)	0.059	34.09 (11.02–105.43)	<0.001
4 to 6	58 (37.4)	97 (60.6)	155 (30.8)	1.43 (0.94–2.19)	0.094	4.08 (1.43–11.64)	<0.05
7 and above	97 (46.2)	113 (53.8)	210 (41.7)	1		1	<0.001

*Marital status*							
Married	95 (25.2)	282 (74.8)	377 (74.8)	19.20 (10.95–33.66)	<0.001	40.35 (14.64–111.18)	<0.001
Single	110 (86.6)	17 (13.4)	127 (25.2)	1		1	

*Educational level*							
Graduate	188 (59.3)	129 (40.7)	317 (62.9)	1		1	
High school or primary school	15 (12.3)	107 (87.7)	122 (24.2)	10.39 (5.79–18.66)	<0.001	2.91 (0.78–10.85)	>0.05
No education	2 (3.1)	63 (96.9)	65 (12.9)	45.90 (11.03–190.96)	<0.001	8.65 (1.18–67.80)	<0.05

*Occupation*							
Govt. or NGO employed	155 (58.5)	110 (41.5)	265 (52.6)	1		1	
Self-employed	44 (32.6)	91 (67.4)	135 (26.8)	2.91 (1.88–4.50)	<0.001	15.43 (4.30–49.14)	<0.001
Farmer/unemployed	6 (5.8)	98 (94.2)	104 (20.6)	23.01 (9.74–54.37)	<0.001	1.97 (0.35–11.08)	>0.05

*Family monthly income*							
10000 and above	78 (86.7)	12 (13.3)	90 (17.9)	1		1	
7000 to 10000	101 (59.1)	70 (40.9)	171 (33.9)	4.50 (2.28–8.89)	<0.001	1.07 (0.40–2.87)	>0.05
5000 to 70000	17 (26.2)	48 (73.8)	65 (12.9)	18.35 (8.07–41.74)	<0.001	6.7 (1.97–22.68)	<0.05
3000 to 5000	6 (6.1)	92 (93.9)	98 (19.4)	99.66 (35.74–277.88)	<0.001	58.29 (14.73–215.03)	<0.001
1000 to 3000	3 (3.8)	77 (96.2)	80 (15.9)	166.83 (45.29–614.45)	<0.001	397.79 (56.22–2814.35)	<0.001

## Data Availability

All data supporting the findings of this study are included within the article. Any additional information can be obtained from the corresponding author upon reasonable request.
